# Identification of Major Psychiatric Disorders From Resting-State Electroencephalography Using a Machine Learning Approach

**DOI:** 10.3389/fpsyt.2021.707581

**Published:** 2021-08-18

**Authors:** Su Mi Park, Boram Jeong, Da Young Oh, Chi-Hyun Choi, Hee Yeon Jung, Jun-Young Lee, Donghwan Lee, Jung-Seok Choi

**Affiliations:** ^1^Department of Psychiatry, SMG-SNU Boramae Medical Center, Seoul, South Korea; ^2^Department of Statistics, Ewha Womans University, Seoul, South Korea; ^3^Department of Psychiatry and Behavioral Science, Seoul National University College of Medicine, Seoul, South Korea; ^4^Institute of Human Behavioral Medicine, Seoul National University Medical Research Center, Seoul, South Korea

**Keywords:** classification, electroencephalography, machine learning, psychiatric disorder, resting-state brain function, power spectrum density, functional connectivity

## Abstract

We aimed to develop a machine learning (ML) classifier to detect and compare major psychiatric disorders using electroencephalography (EEG). We retrospectively collected data from medical records, intelligence quotient (IQ) scores from psychological assessments, and quantitative EEG (QEEG) at resting-state assessments from 945 subjects [850 patients with major psychiatric disorders (six large-categorical and nine specific disorders) and 95 healthy controls (HCs)]. A combination of QEEG parameters including power spectrum density (PSD) and functional connectivity (FC) at frequency bands was used to establish models for the binary classification between patients with each disorder and HCs. The support vector machine, random forest, and elastic net ML methods were applied, and prediction performances were compared. The elastic net model with IQ adjustment showed the highest accuracy. The best feature combinations and classification accuracies for discrimination between patients and HCs with adjusted IQ were as follows: schizophrenia = alpha PSD, 93.83%; trauma and stress-related disorders = beta FC, 91.21%; anxiety disorders = whole band PSD, 91.03%; mood disorders = theta FC, 89.26%; addictive disorders = theta PSD, 85.66%; and obsessive–compulsive disorder = gamma FC, 74.52%. Our findings suggest that ML in EEG may predict major psychiatric disorders and provide an objective index of psychiatric disorders.

## Introduction

As the standard of clinical practice, the establishment of psychiatric diagnoses is categorically and phenomenologically based. According to the International Classification of Disorders (ICD) and the Diagnostic and Statistical Manual for Mental Disorders (DSM) (1, 2), clinicians interpret explicit and observable signs and symptoms and provide categorical diagnoses based on which those symptoms fall into. This descriptive nosology enhances the simplicity of communication; however, it is limited by potentially insufficient objectivity as it relies on observation by the clinician and/or the presenting complaints reported by the patient or informant. In addition, the current system does not encompass psychopathology, in that symptom heterogeneity in the same category of disorder, or homogeneity among other disorders often is present. Research has found that symptom-focused diagnosis limits the focus of treatment to symptom relief only; therefore, data-driven approaches to study neural/biological mechanisms, such as the Research Domain Criteria project by the National Institute of Mental Health, have recently been used as a diagnostic aid (3, 4).

In mental healthcare, advances in data and computational science are rapidly changing. With respect to neural mechanisms and objective markers, the extent of evidence that we can measure has broadened. Additionally, use of machine learning (ML), such as artificial intelligence, has increased. Using out-of-sample estimates, ML can prospectively assess the performance of predictions on unseen data (test data) not used prior to model fitting (training data), thereby providing individualized information and yielding results with a potentially high level of clinical translation (5). This approach is contrary to classical inference based on null hypothesis tests (e.g., *t*-test, analysis of variance), which retrospectively focuses on in-sample estimates and group differences and thus lacks personalized explanation (6). ML is expected to help or possibly replace clinician decisions such as diagnosis, prediction, and prognosis or treatment outcomes (7).

The majority of current neuroimaging research (i.e., using functional magnetic resonance imaging) has applied supervised ML for diagnostic classification between patients and healthy controls (HCs). Studies have predominantly focused on Alzheimer's disease, schizophrenia, and depression (8–10) but have more recently expanded to other diagnostic topics (11). The literature suggests that ML can be used to discriminate psychiatric disorders using brain data with over 75% accuracy (12). A recent review (13) that used a support vector machine (SVM), a common ML method, to assess imaging data found that it is possible to distinguish patients with schizophrenia from HCs as follows: 17 of 22 studies found over 80% accuracy for the classification of validation data and top approaches, respectively (14).

Many imaging studies have compared HCs with subjects with one or several disorders, but few have comprehensively compared many disorders. This may be because acquiring imaging data is associated with high costs, especially when including sufficient patients for each group, a prerequisite for applying any supervised ML algorithm. Another alternative that can measure brain activity is electroencephalography (EEG), which delivers information about voltage measured through electrodes placed on the scalp. EEG is non-invasive, cost-effective, and suitable for measuring resting-state brain activity in natural settings, allowing easy acquisition of large amounts of data. In addition, as the acquisition technology is simplified and the calculation method is advanced, EEG is gaining attention as a core technology of brain–computer interface (BCI). One recent EEG study suggested that EEG spectra ML, using linear discriminant analysis learning method, can discriminate patients with schizophrenia from HCs with an accuracy of 80.66% (15); however, the main trend has been to differentiate between patients with single disorders [e.g., schizophrenia, depression, addiction, and post-traumatic stress disorder (PTSD), and dementia] and HCs (16–19). Notably, EEG features used for classification have differed from study to study; however, EEG studies that include a variety of psychiatric disorders are beginning to emerge (20).

Here, we aimed to establish novel classifiers for discriminating patients with major psychiatric disorders from HCs. We retrospectively collected EEG data of patients with six main categories of psychiatric disorders (i.e., schizophrenia, mood disorders, anxiety disorders, obsessive–compulsive disorders, addictive disorders, and trauma and stress-related disorders) and their specific diagnoses (i.e., depressive and bipolar disorders), excluding neurodevelopmental disorders. To increase the utility of our results, classification models were constructed using spectral power and functional connectivity (FC) features, which are commonly used EEG parameters in clinical settings (21, 22). Selected from ML methods, SVM and random forest (RF) were applied, which have been widely used in various fields of disease diagnosis; however, they struggled to explain the results of the model. Hence, we also performed a penalized logistic regression method, elastic net (EN) (23), to explain the results from the multivariate EEG parameters and facilitate a comparison of major discriminant features between disorders.

## Materials and Methods

### Experimental Subjects

Data were collected retrospectively from medical records, psychological assessment batteries, and quantitative EEG (QEEG) at resting-state assessments from January 2011 to December 2018 from the Seoul Metropolitan Government-Seoul National University (SMG-SNU) Boramae Medical Center in Seoul, South Korea. The original diagnostic decision for clinical patients who visited the medical center was made by a psychiatrist based on DSM-IV or DSM-5 criteria and was also assessed using the Mini-International Neuropsychiatric Interview during psychological assessments. Final clinical confirmation of the primary diagnosis was established by two psychiatrists and two psychologists from March 2019 to August 2019, who reviewed both the original diagnoses in electrical medical records and psychological assessments that had been completed 1 month before and after QEEG. Concurrently, we included a HC sample (*n* = 95), which was selected from the studies performed at the SMG-SNU Boramae Medical Center. The final analyses included 945 subjects. The inclusion criteria were as follows: age from 18 to 70 years; diagnosis of the following primary diagnoses, which fall into six large-category diagnoses and nine specific diagnoses: schizophrenia (*n* = 117), mood disorders [(*n* = 266); depressive disorder (*n* = 119) and bipolar disorders (*n* = 67)], anxiety disorders [(*n* = 107); panic disorder (*n* = 59) and social anxiety disorders (*n* = 48)], obsessive–compulsive disorder (*n* = 46), addictive disorders [(*n* = 186); alcohol use disorder (*n* = 93) and behavioral addiction including gambling and Internet gaming disorders (*n* = 93)], and trauma and stress-related disorders [(*n* = 128); PTSD (*n* = 52), acute stress disorder (*n* = 38), and adjustment disorder (*n* = 38)]; and no difficulty in reading, listening, writing, or understanding Hangeul (Korean language). The exclusion criteria were as follows: lifetime and current medical history of a neurological disorder or brain injury, neurodevelopmental disorder [i.e., intellectual disability [intelligence quotient (IQ) < 70] or borderline intellectual functioning (70 < IQ < 80), tic disorder, or attention deficit hyperactivity disorder), or any neurocognitive disorder. Ethical Approval.

This study was approved by the institutional review board (20-2019-16). In accordance with the retrospective study design, participant consent was waived.

### EEG Settings and Parameters

EEG data included 5 min eyes-closed resting-state with 19 or 64 channels acquired with 500–1,000 Hz sampling rate and 0.1–100 on-line filters *via* Neuroscan (Scan 4.5; Compumedics NeuroScan, Victoria, Australia). Electrode impedances were kept below 5 kΩ by application of an abrasive and electrically conductive gel. In the analysis, the EEG data were down-sampled to 128 Hz, and 19 channels were selected based on the international 10–20 system in conjunction with a mastoid reference electrode as follows: FP1, FP2, F7, F3, Fz, F4, F8, T7, C3, Cz, C4, T8, P7, P3, Pz, P4, P8, O1, and O2. The ground channel was located between the FPz and Fz electrodes. Using the Neuroguide system (NG Deluxe 3.0.5; Applied Neuroscience, Inc., Largo, FL, USA), continuous EEG data were converted into the frequency domain using the fast Fourier transformation (FFT) with the following parameters: epoch = 2 s, sample rate = 128 samples/s (256 digital time points), frequency range = 0.5–40 Hz, and a resolution of 0.5 Hz with a cosine taper window to minimize leakage. Due to the mathematics of the FFT, a single epoch of time will be noisy; we used at least 60 s length of time. Details for EEG pre-processing and artifact rejection are described in a previous study (24) and are also provided in the online supplement. In the current study, power spectral density (PSD; μV2/Hz) and FC were included as EEG parameters. Each EEG parameter was calculated in the following frequency bands: delta (1–4 Hz), theta (4–8 Hz), alpha (8–12 Hz), beta (12–25 Hz), high beta (25–30 Hz), and gamma (30–40 Hz). PSD is the actual spectral power measured at the sensor level, and the absolute power value in each frequency band was included. FC was represented by coherence value, a measure of synchronization between two signals based on phase consistency (25, 26). To minimize the effects of windowing in the FFT (27), an EEG sliding average of the 256-point FFT cross-spectral matrix was computed for each subject. The EEG data were edited by advancing in 64-point steps (75% overlap), recomputing the FFT, and continuing with the 64-point sliding window of the 256-point FFT cross-spectrum for the entire edited EEG record. The mean, variance, standard deviation, sum of squares, and squared sum of the real (cosine) and imaginary (sine) coefficients of the cross-spectral matrix were computed across the sliding average of the edited EEG for all 19 channels for a total number of 81 and 1,539 log-transformed elements for each participant. The following equation was used to calculate coherence (28):

$$coherence\;(f)=\frac{{\left(\sum_{N}(a(x)u(y)+b(x)v(y))\right)}^{2}+{\left(\sum_{N}(a(x)v(y)+b(x)u(y))\right)}^{2}}{\sum_{N}(a{\left(x\right)}^{2}+b{\left(x\right)}^{2})\sum_{N}(u{\left(y\right)}^{2}+v{\left(y\right)}^{2})}$$

and

*a*(*x*) = cosine coefficient for the frequency (*f* ) for channel *x*; *b*(*x*) = sine coefficient for the frequency (*f* ) for channel *x*; *u*(*y*) = cosine coefficient for the frequency (*f* ) for channel *y*; and *v*(*y*) = sine coefficient for the frequency (*f* ) for channel *y*. Supplementary Figures 1–4 provide linked-ear topographic maps for PSD and FC.

### Data Analysis

#### Statistical Analysis

Descriptive statistics were used to examine the overall distribution of the demographic characteristics for each participant (Table 1). To test the difference of demographic variables between each clinical subject and HC, *t*-tests and chi-squared tests were performed for continuous and binary variables, respectively. The patterns of these variables were different between clinical participants, age, sex, and/or years of education; therefore, their effects were included in the model for adjustment in further analyses. Furthermore, IQ is a major psychological variable that can be associated with QEEG (29) and can be considered a result of psychiatric symptoms (i.e., psychomotor retardation). Therefore, subsequent analyses compared models with adjusted and unadjusted IQ. Statistical analyses were conducted using R (version 3.6.3; https://www.r-project.org).

**Table 1:** Demographic characteristics of samples.

**Main/specific**	**Age**		**Sex**		**Education**		**IQ**
	**Mean (SD)**	***t***	**Counts (proportions)**	**χ^**2**^**	**Mean (SD)**	***t***	**Mean (SD)**
Healthy control (*n* = 95)	25.72 (4.55)		Male: 60 (63.2%) Female: 35 (36.8%)		14.91 (2.06)		116.24 (10.94)
Schizophrenia (*n* = 117)	31.73 (12.10)	4.58^***^	Male: 65 (55.6%) Female: 52 (44.4%)	1.25	12.84 (2.95)	−5.76^***^	89.62 (17.51)
Mood disorder (*n* = 266)	30.87 (12.70)	3.86^***^	Male: 151 (56.8%) Female: 115 (43.2%)	1.17	13.31 (2.48)	−5.59^***^	101.58 (15.70)
Depressive disorder (*n* = 199)	31.26 (13.23)	3.96^***^	Male: 109 (54.8%) Female: 90 (45.2%)	1.84	13.05 (2.51)	−6.25^***^	101.85 (15.28)
Bipolar disorder (*n* = 67)	29.71 (11.01)	3.17^**^	Male: 42 (62.7%) Female: 25 (37.3%)	0.00	14.11 (2.21)	−2.36^*^	100.81 (16.98)
Anxiety disorder (*n* = 107)	29.01 (10.56)	2.81^**^	Male: 79 (73.8%) Female: 28 (26.2%)	2,67	13.14 (2.42)	−5.52^***^	98.31 (16.31)
Panic disorder (*n* = 59)	31.05 (11.30)	4.10^***^	Male: 38 (64.4%) Female: 21 (35.6%)	0.25	13.45 (2.91)	−3.62^***^	100.31 (14.77)
Social anxiety disorder (*n* = 48)	26.51 (9.09)	0.69	Male: 41 (85.4%) Female: 7 (14.6%)	7.61^**^	12.78 (1.60)	−6.28^***^	95.85 (17.89)
Obsessive–compulsive disorder (*n* = 46)	28.48 (9.83)	2.28^*^	Male: 38 (82.6%) Female: 8 (17.4%)	5.53^*^	13.93 (2.33)	−2.45^*^	107.80 (15.24)
Addictive disorder (*n* = 186)	29.63 (10.89)	3.34^***^	Male: 164 (88.2%) Female: 22 (11.8%)	24.33^***^	13.23 (2.53)	−5.55^***^	103.88 (16.19)
Alcohol use disorder (*n* = 93)	34.16 (11.88)	6.45^***^	Male: 75 (80.6%) Female: 18 (19.4%)	7.09^**^	13.29 (3.07)	−4.22^***^	103.38 (13.61)
Behavioral addiction disorder (*n* = 93)	25.09 (7.48)	−0.70	Male: 89 (95.7%) Female: 4 (4.3%)	30.26^***^	13.16 (1.89)	−6.02^***^	104.38 (18.49)
Trauma and stress-related disorder (*n* = 128)	36.09 (13.82)	7.03^***^	Male: 44 (34.4%) Female: 84 (65.6%)	18.15^***^	13.57 (2.45)	−4.28^***^	98.89 (15.86)
Post-traumatic stress disorder (*n* = 52)	42.74 (13.0)	11.55^***^	Male: 14 (26.9%) Female: 38 (73.1%)	17.65^***^	13.37 (2.54)	−3.95^***^	98.90 (15.69)
Acute stress disorder (*n* = 38)	28.90 (9.05)	2.69^**^	Male: 3 (7.9%) Female: 35 (92.1%)	33.25^***^	14.26 (2.27)	−1.59	104.06 (15.43)
Adjustment disorder (*n* = 38)	34.19 (14.90)	5.01^***^	Male: 27 (75.0%) Female: 11 (25.0%)	0.74	13.26 (2.41)	−4.21^***^	94.24 (15.41)

* *p < 0.05*,

** *p < 0.01*,

*** *p < 0.001*.

*Statistical values are results from comparison between each psychiatric disorder and healthy controls. IQ, Intelligence Quotient*.

### Classification of Psychiatric Disorders Based on QEEG

#### Feature Combination

**Figure 1:**
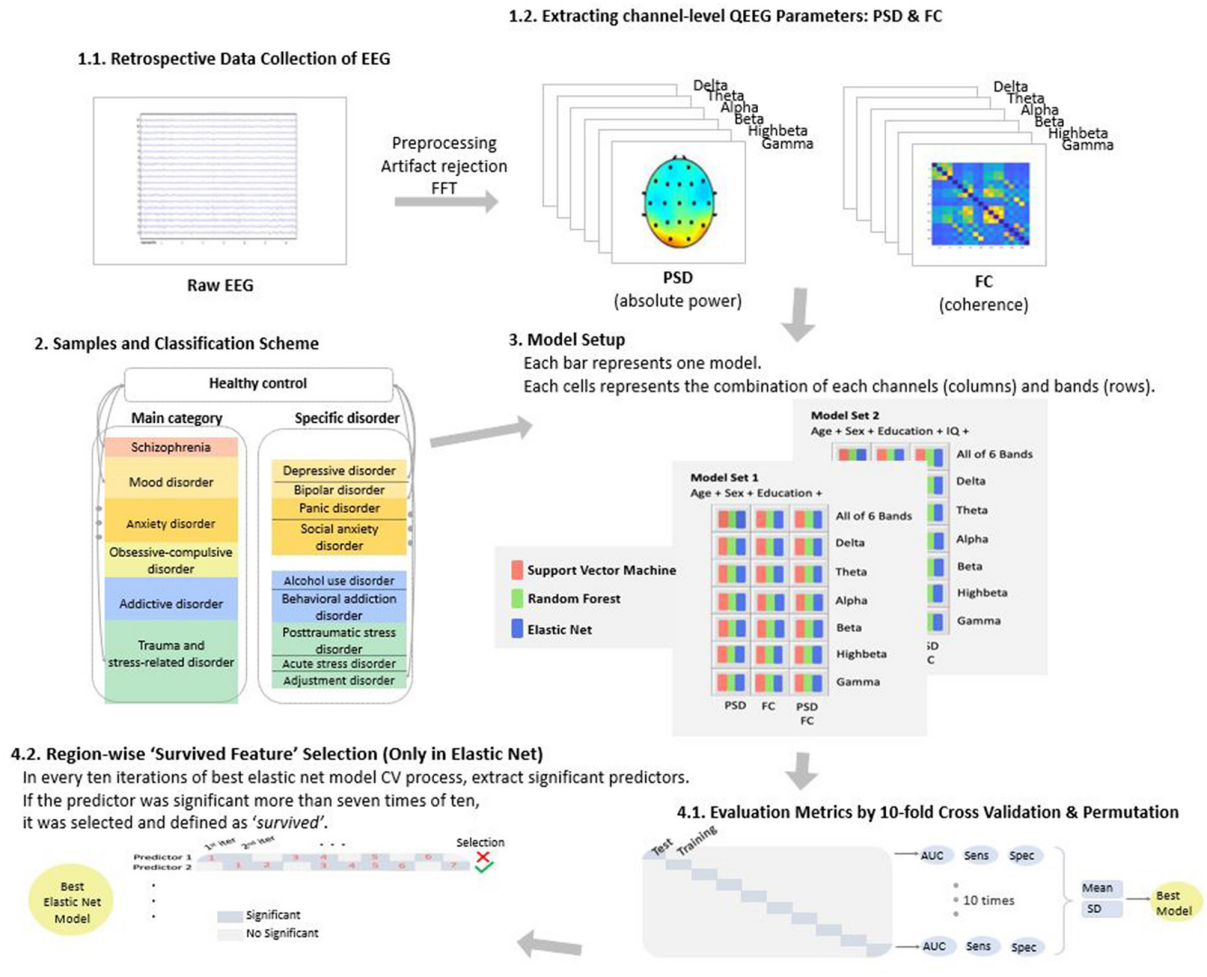
Overview of the study. EEG, electroencephalography; QEEG, quantitative EEG; PSD, power spectrum density; FC, functional connectivity; and FFT, fast Fourier transformation.

For QEEG, feature combinations that were computed in classification models were a mixture of the following conditions: QEEG parameters including PSD (number of features = 19), FC (number of features = 171), and PSD + FC (number of features = 190); QEEG parameters in each frequency band including delta, theta, alpha, beta, high beta, gamma, and all six bands; and adjusting for age, sex, education, and IQ. The number of features computed in the ML model ranged from 22 (i.e., 19 channel PSD in the delta band + age + sex + education) to 1,144 [i.e., (19 channel PSD + 171 pair FC) × all six bands + age + sex + education + IQ; Figure 1].

#### Classification Model

We considered three ML methods for classifying psychiatric disorders: SVM, RF, and logistic regression with EN penalty.

##### SVM

SVM is one of the most frequently used ML methods in binary classification. The main idea of SVM is to find a linear separating hyperplane that maximizes the margin—that is, the largest distance gap between the two group's data points (30). In general, most data cannot be linearly divided, so original data are mapped to a linearly separable high-dimensional space through the so-called kernel trick. The main hyperparameter of SVM is the regularization amount related with the size of the margin. It prevents the model from overfitting and improves the predictability of new data. We determined this hyperparameter with a grid search method, which finds the optimal parameter value in candidates from a grid of parameter values. The range of candidate values was set to (0.1, 0.5, 1, 5, 10). For SVM fitting, we used the R-package *rminer*, which provides various classification and regression methods, including SVM, under the same coherent function structure. In addition, it also allows us to tune the hyperparameters of the models.

##### RF

RF (31) is based on an ensemble technique that makes better predictions by combining multiple decision trees. The performance of a single decision tree is unstable since the generated decision trees differ according to the training dataset. To handle this problem, RF uses a bagging technique that builds many trees that randomly extract only some features and averages them. RF generally has a high level of performance by reducing the variance of prediction compared to that of a single model. The main hyperparameter of RF is the number of features randomly extracted when building a tree. Following Hastie et al. (32) we used the default value for the classification problem: the squared root of the total number of features. We used the R-package *random Forest*, which allows to fit RF by performing classification based on a forest of trees using random inputs and can examine the importance of each variable in the created model.

##### EN

When applying logistic classification with high-dimensional features, penalized logistic classification is commonly used for avoiding the ill-posed problem. Among the various penalty terms, the EN introduced by Zou and Hastie (23) works well when the input features are strongly correlated. Because the EN penalty is a compromise of the ridge and lasso penalty, it can effectively select the relevant variables and encourage highly correlated variables to be averaged. EN has a hyperparameter that indicates the amount of penalty used in the model. The optimal value of λ was selected by *K*-fold cross-validation. The R-package *glmnet* was used for fitting EN. *glmnet* is a package that fits classification or regression models *via* penalized maximum likelihood. It can handle lasso, EN, and ridge penalty through the regularization parameter λ; it provides the fast automatic search algorithm for finding the optimal value of λ.

*Cross-Validation and Feature Extraction*. The performance of all models was compared based using 10-fold cross-validation, which partitions the original sample into 10 disjointed subsets, using nine of those subsets in the training process, and then making predictions about the remaining subset. Furthermore, for each fold, EN extracts the relevant features, which have non-zero estimates of regression coefficients. If the estimates of a feature were not zero more than seven times among 10-fold groups, we considered the feature to have “survived.”

*Permutation Test*. We conducted a permutation test to assess the significance of each of the best EN models. We generated 1,000 random permutations and constructed the null distribution of the area under curve (AUC) (33, 34). *p*-values were obtained by calculating the number of cases that exceeded the AUC of the best EN model.

## Results

### Comparison of Models

To select the model, we compared the performance of SVM, RF, and EN in terms of AUC. Regardless of adjusting for IQ, the accuracies of SVM, RF, and EN were each above the level of chance. With respect to the prediction of distinguishing patients with main-categorical psychiatric disorders from the HCs, EN showed the highest accuracy, in that the mean AUC for all disorders adjusted for IQ was 87.59 ± 7.92% (SVM = 86.02 ± 8.89% and RF = 87.18 ± 8.08%). EN also demonstrated the highest mean AUC performance for specific disorders (EN = 87.76 ± 8.42%, SVM = 82.83 ± 7.62%, and RF = 86.16 ± 8.97%). Therefore, EN was selected as the final method for further analyses. Supplementary Tables 1, 2 show results for the comparisons of SVM, RF, and EN in detail.

Tables 1, 2 show the EN results of the discrimination model and feature combinations for each type of disorder. Compared to all features, PSD + FC in all bands were added to the models and select features showed superior classification accuracy (Tables 1, 2). In addition, adjusting IQ enhanced the performance of discrimination models compared to leaving IQ unadjusted (Supplementary Figure 5).

**Table 2:** Comparison of elastic net models in predicting outcomes in patients with psychiatric disorders distinguished from healthy controls in major category diagnoses.

	**Feature**	**AUC**	**Sens**	**Spec**	**Feature**	**AUC**	**Sens**	**Spec**	***p*** **-value** **(best)**
	**(Entire)**	**Mean (SD)**			**(best)**	**Mean (SD)**			
Schizophrenia	Entire	87.08	85.11	85.30	Alpha PSD	93.83	91.44	92.42	<0.001
		(5.48)	(10.57)	(12.15)		(5.74)	(9.88)	(10.73)	
Mood disorder	Entire	84.98	85.44	78.91	Theta FC	89.26	89.33	80.85	<0.001
		(5.04)	(11.25)	(13.38)		(6.20)	(11.50)	(12.19)	
Anxiety disorder	Entire	88.95	82.00	91.33	Whole PSD	91.03	83.18	91.78	<0.001
		(6.47)	(13.30)	(10.17)		(5.29)	(11.99)	(6.42)	
Obsessive–compulsive disorder	Entire	65.00	59.78	85.00	Gamma FC	74.52	65.33	90.00	0.005
		(17.40)	(17.91)	(19.44)		(18.43)	(22.10)	(21.60)	
Addictive disorder	Entire	76.70	70.58	83.67	Theta PSD	85.66	71.61	94.89	<0.001
		(10.04)	(17.25)	(19.84)		(5.22)	(12.45)	(5.40)	
Trauma and stress-related disorder	Entire	86.52	87.67	81.99	Beta FC	91.21	86.44	90.64	<0.001
		(10.09)	(15.45)	(11.21)		(5.30)	(9.58)	(6.04)	

*Age, sex, education, and IQ were included in the model. Permutation test was conducted in order to assess the significance of each of the best EN models. EN, elastic net; PSD, power spectrum density; FC, functional connectivity; IQ, intelligence quotient*.

### Best Feature Combinations

All best models for each disorder from EN significantly distinguished between patients with psychiatric disorders and HCs (*p* < 0.05). The best feature combinations and classification accuracies for discrimination between patients with each large-category of diagnosis and HCs with adjusted IQ were as follows (Table 2): schizophrenia = alpha PSD, 93.83 ± 5.74%; trauma and stress-related disorders = beta FC, 91.21 ± 5.30%; anxiety disorders = whole band PSD, 91.03 ± 5.29; mood disorders = theta FC; 89.26 ± 6.20; addictive disorders = theta PSD, 85.66 ± 5.22; and obsessive–compulsive disorder = gamma FC, 74.52 ± 18.43. Higher accuracies of best models were found for specific diagnoses, compared to the large diagnostic category. Particularly, the maximum accuracy reached a fairly good level in that the accuracy for PTSD was 95.38 ± 4.90%.

Moreover, the best feature appeared differently based on specific diagnosis, even for those in the same category. The best accuracies for specific disorders after adjusting for IQ were as follows (Table 3): PTSD = beta PSD, 95.38 ± 4.90; adjustment disorder = alpha FC, 93.75 ± 7.31; acute stress disorder = beta PSD + FC, 92.00 ± 6.63; alcohol use disorder = whole band PSD, 93.21 ± 6.31; behavioral addiction = delta PSD, 84.78 ± 8.85; bipolar disorder = delta PSD + FC, 92.13 ± 3.01; depressive disorder = delta FC, 87.92 ± 5.67; social anxiety disorder = theta FC, 90.63 ± 8.51; and panic disorder = whole band PSD, 90.07 ± 5.32.

**Table 3:** Comparison of models in predicting outcomes in patients with psychiatric disorders distinguished from healthy controls in specific diagnoses.

	**Feature**	**AUC**	**Sens**	**Spec**	**Feature**	**AUC**	**Sens**	**Spec**	***p*** **-value** **(best)**
	**(Entire)**	**Mean (SD)**			**(Best)**	**Mean (SD)**			
Depressive disorder	Entire	83.52	68.32	94.89	Delta FC	87.92	80.82	91.44	<0.001
		(10.02)	(16.05)	(8.58)		(5.67)	(15.10)	(12.35)	
Bipolar disorder	Entire	88.3	92.62	79.22	Delta PSD+FC	92.13	90.71	85	<0.001
		(7.62)	(12.32)	(11.93)		(3.01)	(11.25)	(10.76)	
Panic disorder	Entire	88.8	92.78	81	Whole PSD	90.07	89.44	88	<0.001
		(6.85)	(8.34)	(13.34)		(5.32)	(11.04)	(11.46)	
Social anxiety disorder	Entire	84.76	84.11	87.5	Theta FC	90.63	91.56	88	<0.001
		(11.99)	(10.53)	(19.61)		(8.51)	(6.5)	(13.98)	
Alcohol use disorder	Entire	84.04	84.67	82.22	Whole PSD	93.21	92.33	88.44	<0.001
		(9.63)	(20.48)	(13.71)		(6.31)	(7.45)	(13.62)	
Behavioral addiction disorder	Entire	69.6	67.56	79.33	Delta PSD	84.78	81.33	83.67	<0.001
		(10.88)	(19.26)	(25.76)		(8.85)	(13.87)	(14.34)	
Post-traumatic stress disorder	Entire	86.24	79.22	92.33	Beta PSD	95.38	95.88	92	<0.001
		(10.18)	(18.36)	(13.7)		(4.9)	(7.1)	(10.32)	
Acute stress disorder	Entire	87.18	96.66	80.77	Beta PSD+FC	92	95	89.44	<0.001
		(16.04)	(10.54)	(24.56)		(6.63)	(10.54)	(11.27)	
Adjustment disorder	Entire	86.4	92.5	82	Alpha FC	93.75	95	91.66	<0.001
		(10.16)	(16.87)	(11.76)		(7.31)	(10.54)	(13.42)	

*Age, sex, education, and IQ were included in the model. Permutation test was conducted in order to assess the significance of each of the best EN models. EN, elastic netl; PSD, power spectrum density; FC, functional connectivity; IQ, intelligence quotient*.

**Figure 2:**
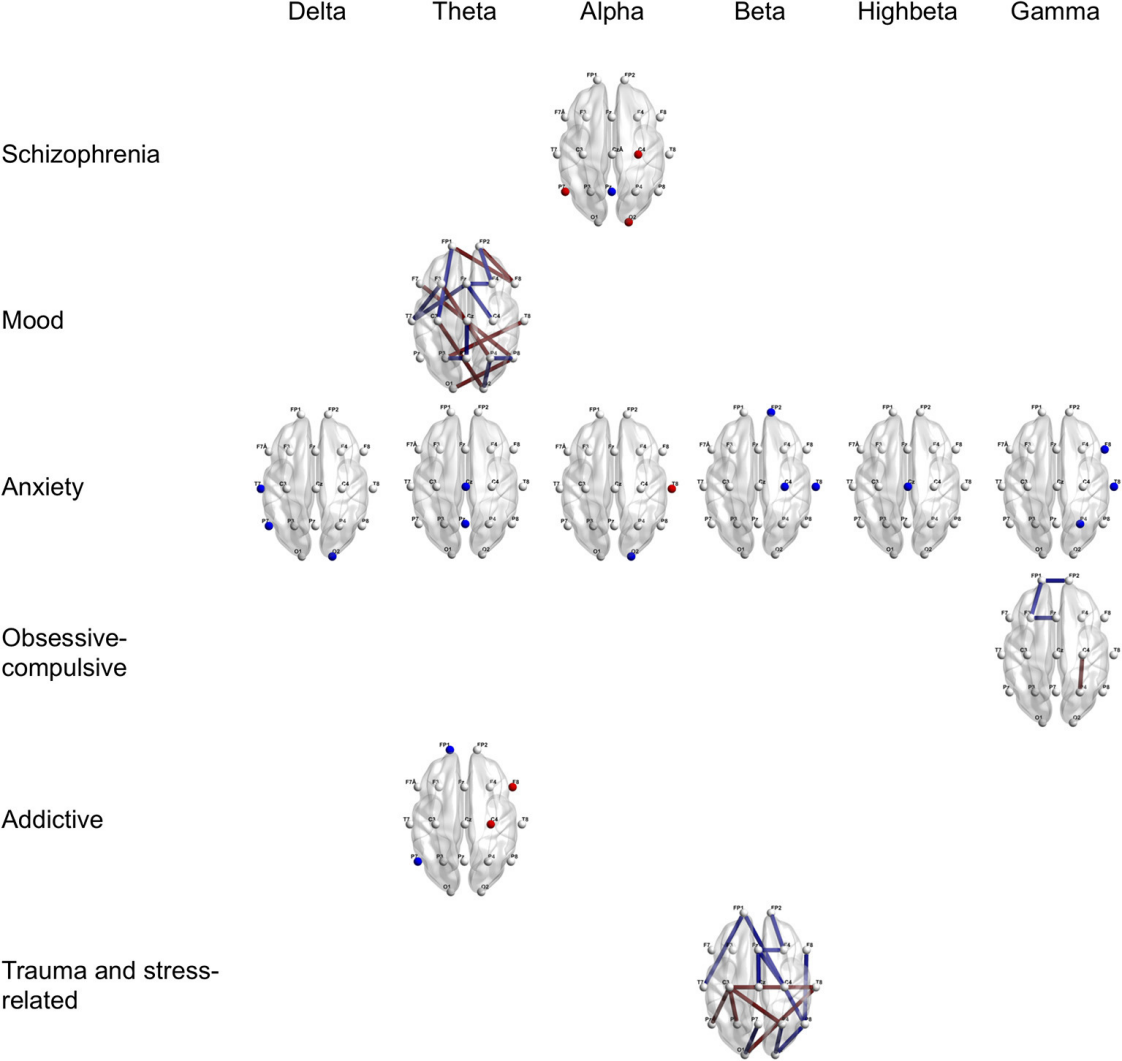
Region-wise predictions for main categories of psychiatric disorders distinguished from healthy controls. Dots and lines represent region-wise survived predictors in the best EN model, which emerged significant above 7 times during 10 times of cross-validation. Dots mean channel-level PSD (absolute power) as QEEG parameter and lines mean channel-level FC (coherence) as QEEG parameter. Features colored in red represent higher probabilities for the psychiatric disorder when more increase. Features colored in blue represent higher probabilities for the psychiatric disorder when more decrease. Age, sex, the year of education, and the IQ score were computed in the models. EN, Elastic Net; PSD, Power Spectrum Density; FC, Functional Connectivity; QEEG, Quantitative Electroencephalography; and IQ, Intelligence Quotient.

**Figure 3:**
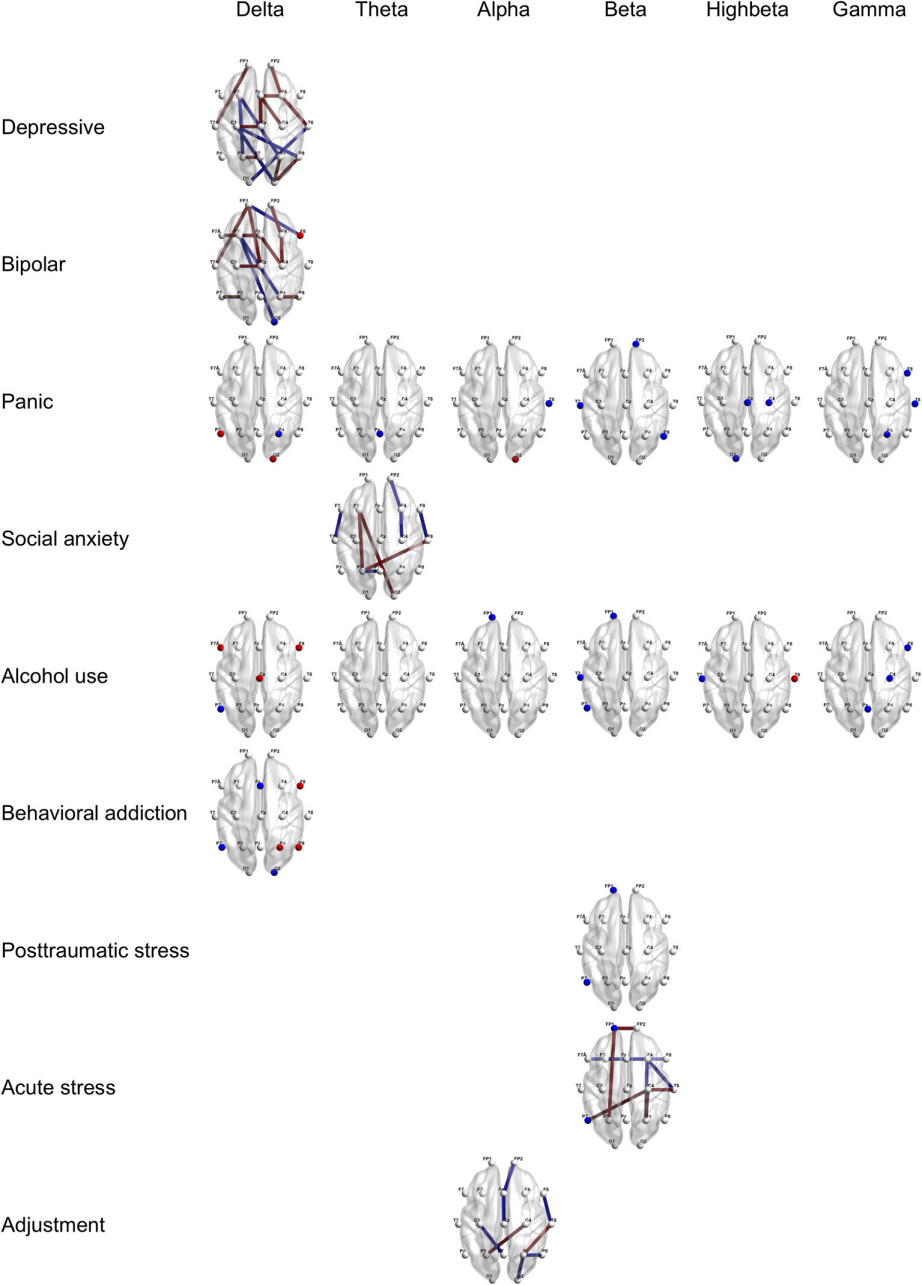
Region-wise predictions for specific psychiatric disorders. Dots and lines represent region-wise survived predictors in the best EN model, which emerged significant above 7 times during 10 times of cross-validation. Dots mean channel-level PSD (absolute power) as QEEG parameter and lines mean channel-level FC (coherence) as QEEG parameter. Features colored in red represent higher probabilities for the psychiatric disorder when more increase. Features colored in blue represent higher probabilities for the psychiatric disorder when more decrease. Age, sex, the year of education, and the IQ score were computed in the models. EN, Elastic Net; PSD, Power Spectrum Density; FC, Functional Connectivity; QEEG, Quantitative Electroencephalography; and IQ, Intelligence Quotient.

Furthermore, Figures 2, 3 provide region-wise predictors in the best EN model that were at a survival rate above 70% during the cross-validation (for more details, see Supplementary Tables 3, 4).

## Discussion

Our current study offers the following clinical insights: higher severity disorders increase the accuracy of the ML discrimination (e.g., classification of schizophrenia demonstrated the best accuracy); classifications for specific diagnoses (e.g., PTSD and acute stress disorder) provide higher accuracy than grouping large categories (e.g., trauma and stress-related disorders); and each disorder classification model shows different EEG characteristics.

First, consistent with our findings, previous imaging studies have found higher diagnostic accuracy for schizophrenia (92%) than bipolar disorder (79%) (35); however, the authors suggest that this may be due to the fact that although both disorders are associated with altered brain activity in several overlapping regions, the magnitude of dysfunction was more pronounced in schizophrenia. Moreover, in the present study, trauma- and stress-related disorders ranked second for accuracy with an AUC of 91.21% among large-category disorders and PTSD ranked first with an AUC of 95.38% among specific diagnoses. Similarly, one study found higher accuracy for PTSD-HC (80.00%) than for major depression-HC (67.92%) discrimination (36). While it is difficult to determine the severity of the disorder by the accuracy alone, it is plausible that functional brain alterations in specific disorders, such as schizophrenia, representative psychiatric disorders, and PTSD with an explicit traumatic event, are more pronounced than that of other psychiatric disorders. Alternatively, the homogeneity of neurodynamical states of intra-diagnostic disorders might influence the accuracy.

Second, we obtained higher accuracy in the specific categories than in the large grouping categories. In particular, in addictive disorders, when alcohol use disorder (93.21%) and behavioral addiction (84.78%) were classified rather than assessed as a large categorical diagnosis (85.78%), the accuracies were much higher. Results of repeated studies of that behavioral addiction, including Internet gaming disorder, have distinguished functional brain features from those in substance abuse disorders (37). With respect to bipolar and depressive disorders, the two were divided into different categories in DSM-5, but in the previous version of DSM and the current version of ICD, they were classified into one (i.e., mood or affective disorder). Compared to the group of mood disorders (89.26%), bipolar disorder showed higher accuracy (92.13%) when classified alone, but the accuracy of depressive disorder (87.92%) was relatively low. These findings may supplement attempts to discriminate mood disorders (38, 39). However, it should be avoided to interpret it as a more serious disease because the accuracy of bipolar disorder was higher than that of depressive disorder. This is because, as mentioned above, neurodynamical state heterogeneity can exist even within the same category. In addition, since several complex factors such as duration of disorder, recurrence, comorbidity, severity of symptoms, and psychotropic medication can affect brain function and EEG (40, 41), the results of this study are not considered to be more discriminatory than HC or inter-disease severity. It is not appropriate to interpret the results of this study by simplifying as that such disease category is better discriminated than the healthy individuals or that there is a hierarchical hierarchy of diseases.

Third, each classification model provides different best predictive features; different EEG patterns may imply the likelihood of diagnosis of distinguished psychiatric disorders. For instance, schizophrenia is best distinguished from HCs by abnormal alpha band power spectra; however, anxiety disorders are best distinguished from HCs by abnormal whole band power spectra. Several key features including beta power abnormalities in trauma and stress-related disorder, theta connectivity abnormalities in social anxiety disorder, and prefrontal connectivity abnormalities in fast frequency in obsessive–compulsive disorder are consistent with previous studies using group difference statistics (21, 42–44). In addition, dysfunctional connectivity in slow frequency bands in depressive disorder has been confirmed by a previous ML study (17). These differences in EEG patterns were also present within the same category (e.g., panic disorder vs. social anxiety disorder; PTSD vs. acute stress disorder). This implies that there is heterogeneity between disorders classified into the same category. In fact, not all patients with acute stress disorder develop PTSD. In this context, one study suggested that fear inhibition was different between acute stress disorder and PTSD groups (45). The key EEG features of each disorder suggested in the present study can provide useful information for diagnostic decisions of individuals in clinical settings. Nevertheless, cautious interpretation of the findings should be implemented, in that key features are not to be considered as directly reflecting the brain mechanisms of the disorder.

This study focused on classification between patients with each mental disorder and HCs. We additionally performed analyses using EN ML between several psychiatric disorders. As in the previous analysis, the effects of demographic data and IQ were treated as covariates. The best EEG feature combination and AUC for each disease discrimination emerged as follows (see Supplementary Table 5 for more details): schizophrenia vs. bipolar disorder = alpha PSD + FC, 67.84 ± 13.67%; schizophrenia vs. mood disorder = alpha PSD, 68.08 ± 7.23%, schizophrenia vs. depressive disorder = theta FC, 68.70 ± 12.67%; bipolar disorder vs. depressive disorder = alpha PSD + FC, 67.84 ± 13.67%; and panic disorder vs. social anxiety disorder = alpha PSD + FC, 70.47 ± 20.91%. Although the results had lower AUC than the comparison between patients with each disorder and HCs, all permutation results showed a higher level of discrimination than chance (*p*s < 0.05). In other words, EEG ML might be helpful for diagnostic decision between psychiatric disorders and HC and also between disorders. Multi-class ML method approach attempts in future studies would enhance the usability of EEG ML.

There is a wide variety of methods for extracting features including time series domain and frequency domain, and methods are still being developed. Extracting relevant features for modeling is crucial for ML to perform dimensional reduction and increase prediction accuracy (46). The current study used channel-level resting-state EEG absolute power as a representative PSD and coherence as the index of FC. These two parameters are where bandwidth field knowledge and research results have been accumulated for several decades (21, 47, 48). Our results can be extended to diagnostic information and help individualized treatment choices. Previous research has reported promising outcomes for predicting treatment responses, including medication and transcranial direct current stimulation, with ML using pre-treatment resting-state EEG (49, 50). In addition, the task-free and resting-state method during acquisition of EEG involves less measurement time than the paradigm using experimental stimulation; thus, it has high accessibility and scalability.

The current study has several limitations. First, the effects of medication, comorbidity, and severity of disorder were not controlled. Second, diagnoses were made near the time EEGs were measured, and we therefore cannot rule out the possibility of mixed results of patients who were subsequently diagnosed with different disorders. Third, the sample is from one center and the race and nationality are limited to Asian and Korean. Finally, our design is retrospective, and we did not prospectively verify the modeling. Moreover, external validation was not performed on other samples. Therefore, for generalization, it is necessary to verify the results with additional samples.

In conclusion, we found that an ML approach using EEG could predict major psychiatric disorders with differing degrees of accuracy according to diagnosis. Each disorder classification model demonstrated different characteristics of EEG features. EEG ML is a promising approach for the classification of psychiatric disorders and has the potential to augment evidence-based clinical decisions and provide objectively measurable biomarkers. It would be advantageous to provide the automated diagnostic tools in future medical healthcare using BCI.

## Data Availability Statement

The datasets presented in this study can be found in online repositories. The names of the repository/repositories and accession number(s) can be found at: https://osf.io/8bsvr/.

## Ethics Statement

The studies involving human participants were reviewed and approved by the Institutional Review Board of SMG-SNU Boramae Medical Center, Seoul, Republic of Korea. The Ethics Committee waived the requirement of written informed consent for participation.

## Author Contributions

SP performed data query and integration, EEG data analysis, statistical modeling, and interpretation of results, and contributed to writing the manuscript. BJ contributed to statistical modeling, programming, and writing the manuscript. DO contributed to data collection and writing the manuscript. C-HC, HJ, and J-YL contributed to data collection and reviewing the manuscript. DL and J-SC contributed to the conceptual design of the study and writing the manuscript. All authors contributed to the article and approved the submitted version.

## Conflict of Interest

The authors declare that the research was conducted in the absence of any commercial or financial relationships that could be construed as a potential conflict of interest.

## Publisher's Note

All claims expressed in this article are solely those of the authors and do not necessarily represent those of their affiliated organizations, or those of the publisher, the editors and the reviewers. Any product that may be evaluated in this article, or claim that may be made by its manufacturer, is not guaranteed or endorsed by the publisher.
