# Supplementary material for: Identification of Major Psychiatric Disorders From Resting-State Electroencephalography Using a Machine Learning Approach
Source: Front Psychiatry. 2021 Aug 18;12:707581. doi: 10.3389/fpsyt.2021.707581 (PMC8416434; doi:10.3389/fpsyt.2021.707581)
Supplement: Supplementary file 1 [file Data_Sheet_1.pdf]

Online Supplement for

Identification of major psychiatric disorders from resting-state electroencephalography using a machine learning approach

Su Mi Park, Boram Jeong, Da Young Oh, Chi-Hyun Choi, Hee Yeon Jung, Jun-Young Lee, Donghwan Lee*, Jung-Seok Choi*

***Correspondence:**

Jung-Seok Choi: 20, Boramae-ro 5-gil, Dongjak-gu, Seoul 07061, South Korea; Tel.: +82-2-870-3461; E-mail: choijs73@gmail.com

Donghwan Lee: 52, Ewhayeodae-gil, Seodaemun-gu, Seoul 03760, South Korea; Tel.: +82-2-3277-2300; E-mail: donghwan.lee@ewha.ac.kr

This file includes:

Materials and Methods

Supplementary References

Figure S1 to S5

Table S1 to S5

METHODS and MATERIALS

EEG data pre-preprocessing

EEG data pre-processing was conducted by the NG Deluxe 3.0.5 system. Digital EEG data importation involves the following steps: 1) down-sampling to 128 Hz; 2) finding the baseline EEG by filtering at <1 Hz and >40 Hz (using 5th order Butterworth filters and creating values from zero time to negative time to allow the filter to start at time point 0); and 3) after finding the baseline EEG, filtering the spliced selections of EEG a second time at <1 Hz and >40 Hz. NeuroGuide uses a splicing method of appending edited selections of EEG (minimum segment length = 600 ms) and then baselines using a Butterworth high-pass filter at 1 Hz and a low-pass filter at 55 Hz so as to minimize splicing artifacts. Next, 19 of the 64 channels with the linked-ear reference were selected for the analysis based on the international 10-20 system: FP1, FP2, F7, F3, Fz, F4, F8, T3, C3, Cz, C4, T4, T5, P3, Pz, P4, T6, O1, and O2.

Artifacts due to eye blinks, movements, and drowsiness during EEG recording were eliminated by visual inspection and the automatic NG Deluxe 3.0.5 cleaning system (NG Deluxe 3.0.5, Applied Neuroscience; St. Petersburg, FL, USA). "Artifact Rejection" and "Generate Edits" options were used to obtain artifact-free selections. The default option for eye movement and drowsiness selection is "High," which is the most sensitive setting, and 1.00 for the "Amplitude Multiplier," which means that the template matches one-to-one to the root-mean-square amplitude of the EEG recording. If the root-mean-square amplitude is equal to or less than the root-mean-square value of the template, then a selection is made. Then, continuous EEG data were converted into the frequency domain using the Fast Fourier transformation (FFT) with the following parameters: epoch = 2 s, sample rate = 128 samples/s (256 digital time points), frequency range = 0.5–40 Hz, and a resolution of 0.5 Hz with a cosine taper window to minimize leakage. It is a well-known fact that when using the Fast Fourier Transform (FFT) that the epoch length determines the frequency resolution. For example, the frequency resolution of a 1 s epoch length = 1 Hz, the frequency resolution of a 2s epoch length = 0.5

Hz, the frequency resolution of a 4 s = 0.25 Hz, etc (Otnes and Enochson, 1978). Due to the mathematics of the FFT a single epoch of time will be noisy, we used at least 60 s length of time.

In the current study, channel-level power spectral density (PSD) was represented by absolute power and functional connectivity (FC) was represented by coherence value, a measure of synchronization between two signals based on phase consistency (Guevara MA &Corsi-Cabrera 1996; Nunez et al. 1999). Each EEG parameter was calculated in the following frequency bands: delta (1-4 Hz), theta (4-8 Hz), alpha (8-12 Hz), beta (12-25 Hz), high beta (25-30 Hz), and gamma (30-40 Hz). Supplementary Figures 1, 2, 3, and 4 provide linked-ear topomaps for PSD and FC.

SUPPLEMENTARY REFERENCES

Guevara MA, Corsi-Cabrera M. 1996. EEG coherence or EEG correlation? Int J Psychophysiol. 23:145-153.

Nunez PL, Silberstein RB, Shi Z, et al. 1999. EEG coherency II: experimental comparisons of multiple measures. Clin Neurophysiol. 110:469-486.

Otnes, R.K. and Enochson, L. 1978. Applied Time Series Analysis. John Wiley & Sons, New York

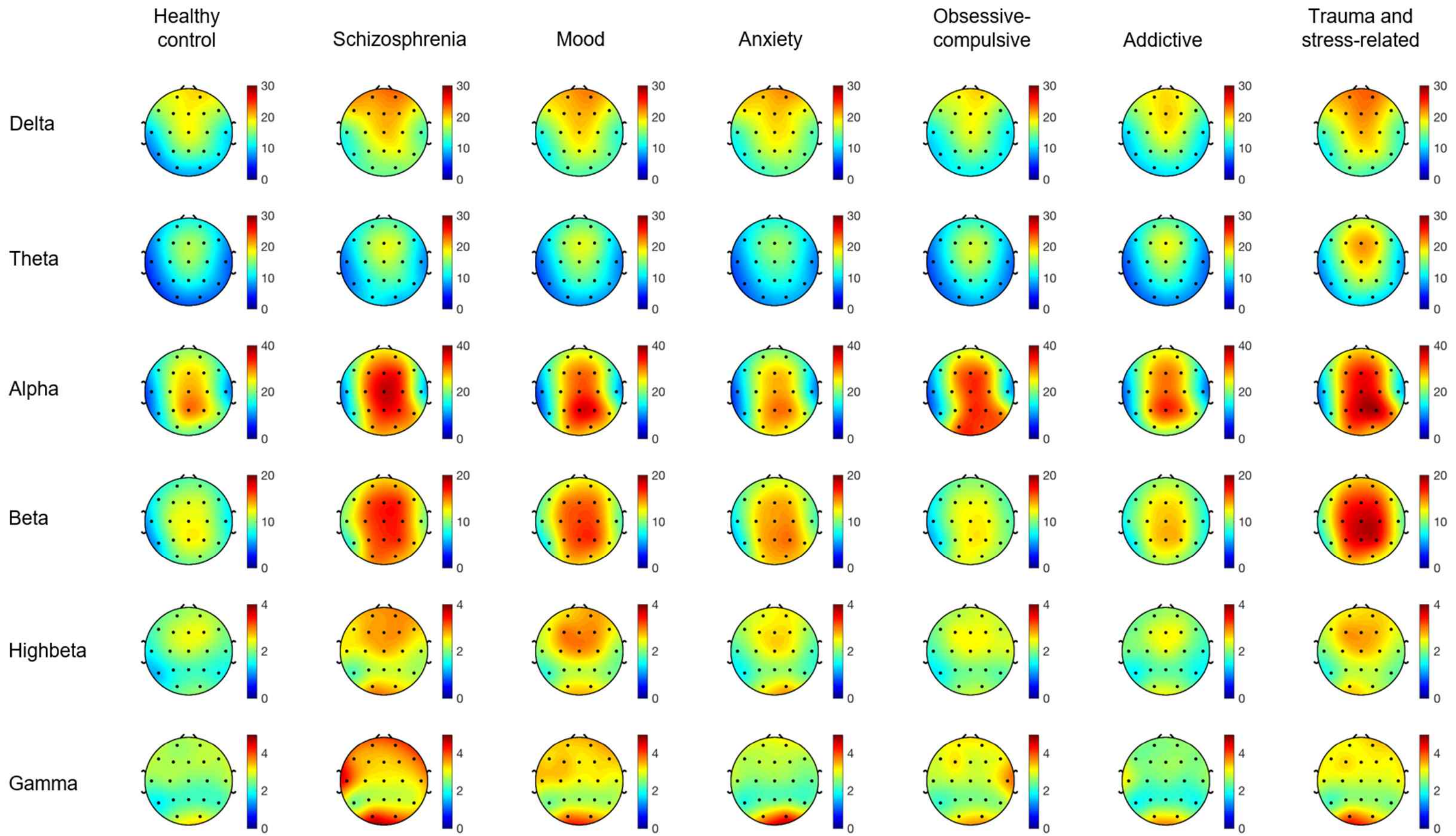

Figure S1. Group mean topoplots for PSD from linked-ear reference QEEG for main category of mental disorders and HCs in each frequency band. PSD means absolute power of QEEG. PSD = Power Spectrum Density, QEEG = Quantitative Electroencephalography, and HC = Healthy Controls.

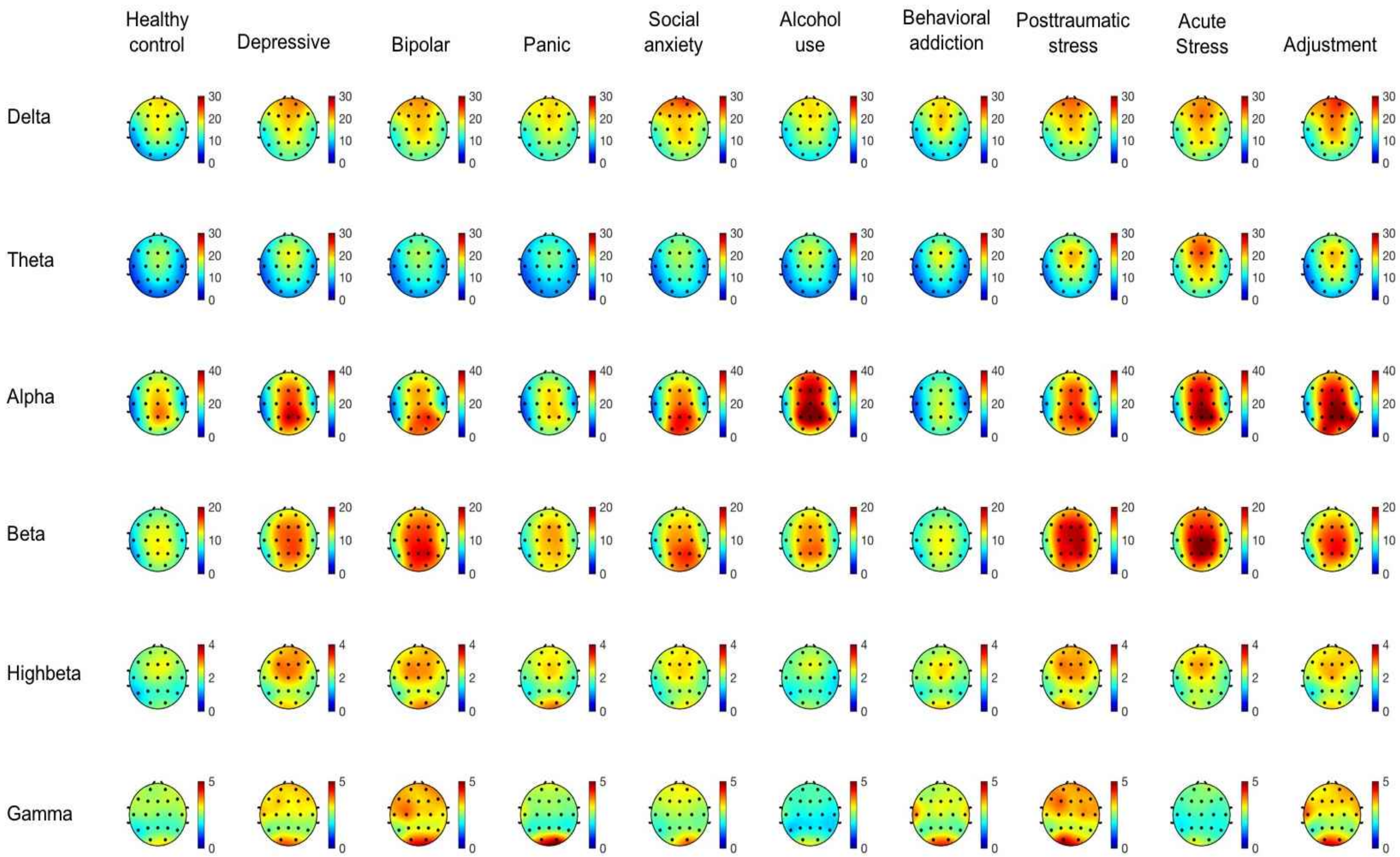

Figure S2.Group mean topoplots for PSD from linked-ear reference QEEG for specific mental disorders and HCs in each frequency band.PSD means absolute power of QEEG. PSD = Power Spectrum Density, QEEG = Quantitative Electroencephalography, and HC = Healthy Controls.

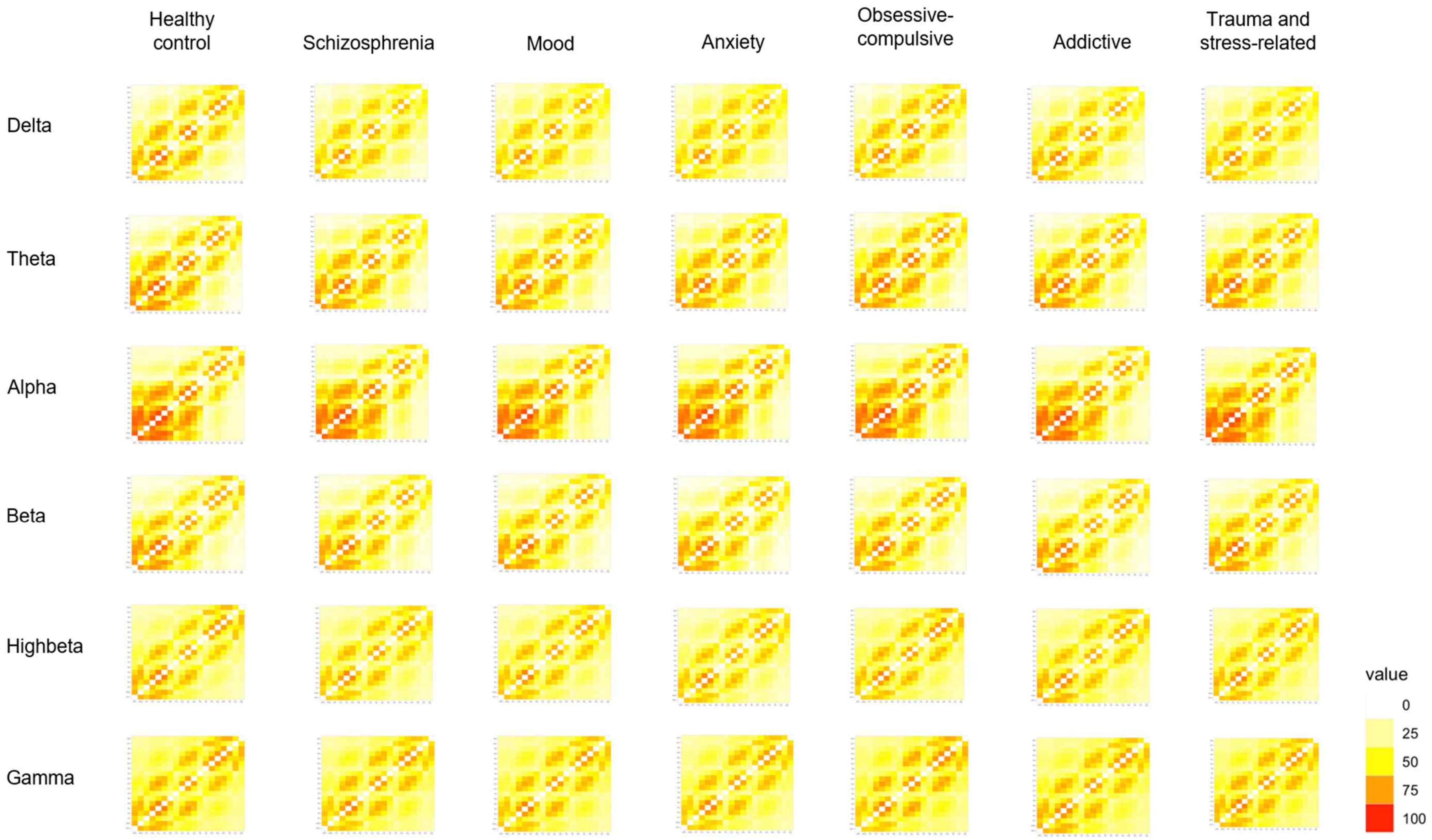

Figure S3. Group mean heatmap for FC from linked-ear reference QEEG for main category of psychiatric disorders and HCs. FC means coherence of QEEG, FC = Functional Connectivity, QEEG = Quantitative Electroencephalography, and HC = Healthy Controls.

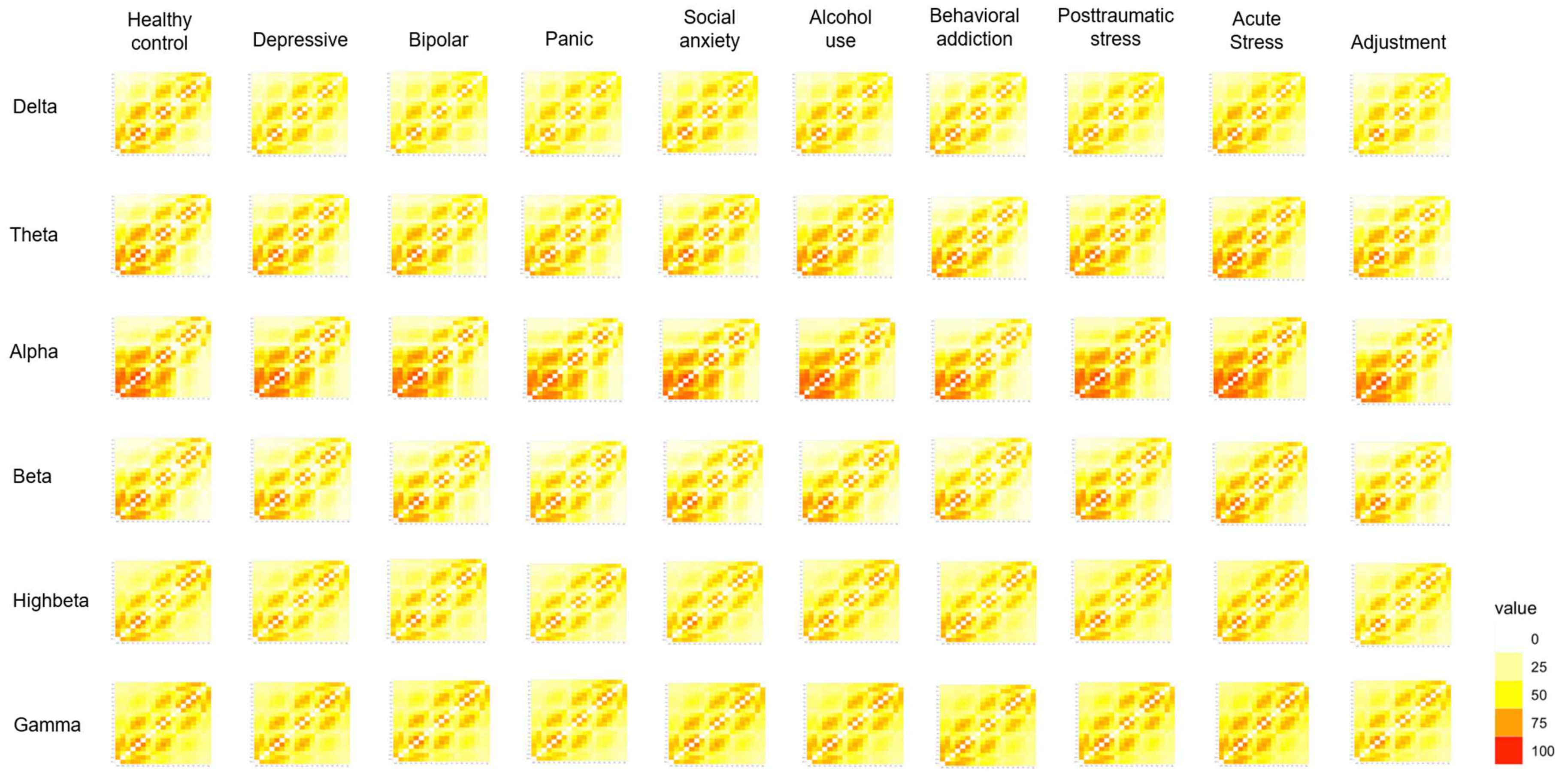

Figure S4. Group mean heatmap for FC from QEEG for specific psychiatric disorders and HCs. FC means coherence of QEEG, FC = Functional Connectivity, QEEG = Quantitative Electroencephalography, and HC = Healthy Controls.

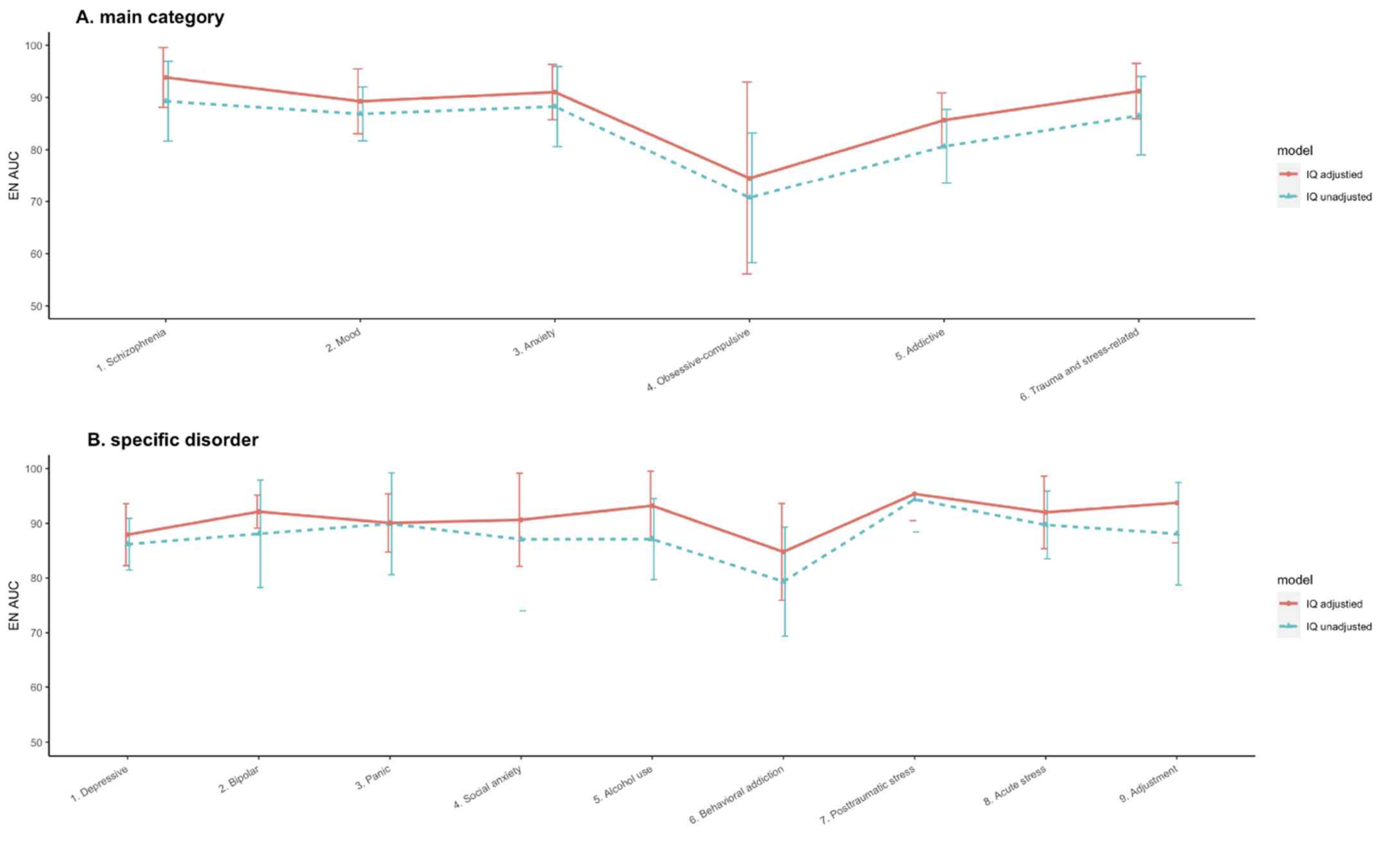

Figure S5. Group mean AUC for the best combination of features in EN models for distinguishing patients with psychiatric disorders from healthy controls Age, sex, and the year of education were computed in the models. 10-fold cross validation was applied.EN = Elastic Net, AUC = Area Under Curve, and IQ = Intelligence Quotient.

Table S1. Comparisons of AUC performance of classification models for main category of psychiatric disorders distinguished from HCs

			**Predictor: EEG+age+sex+education**				**Predictor: EEG+age+sex+education+IQ**		
			AUC	Specificity	Sensitivity		AUC	Specificity	Sensitivity
**Main Category of disorder**	**ML method**	**Best feature**		Mean(SD)		**Best feature**		Mean(SD)	
Schizosphrenia	SVM	Theta PSD+FC	85.5 (6.7)	81.2 (9.6)	84.5 (11.5)	Theta PSD	91.4 (4.7)	92.7 (6.9)	85.6 (10.4)
	RF	Delta FC	86.08 (8.25)	85.22 (12.74)	82.95 (15.87)	Theta FC	93.8 (4.35)	90.11 (14.29)	90.68 (9.16)
	EN	Theta FC	89.28 (7.63)	89.33 (11.04)	85.61 (14.2)	Alpha PSD	93.83 (5.74)	91.44 (9.88)	92.42 (10.73)
Mood disorder	SVM	Delta PSD+FC	84.3 (5.8)	83 (11.6)	81.1 (12.3)	Delta FC	87.3 (3.6)	82.9 (14.6)	83.2 (10.9)
	RF	Delta PSD+FC	83.57 (5.8)	83.11 (14.02)	80.03 (10.68)	Theta FC	85.57 (4.63)	83.22 (12.19)	77.74 (14.73)
	EN	Delta PSD+FC	86.85 (5.16)	85.44 (12.2)	81.23 (10.45)	Theta FC	89.26 (6.2)	89.33 (11.5)	80.85 (12.19)
Anxiety disorder	SVM	Delta FC	85.8 (6.1)	84.9 (12.2)	85.1 (14.2)	Delta FC	87.6 (9.5)	78.5 (15.9)	90.7 (11.3)
	RF	Theta PSD+FC	87.02 (7.18)	85.73 (15.58)	83.44 (10.9)	Delta PSD+FC	90.08 (6.78)	84.27 (10.55)	91.78 (8.29)
	EN	Delta PSD+FC	88.26 (7.67	82.09 (14.3)	89.22 (12.84)	Whole PSD	91.03 (5.29)	83.18 (11.99)	91.78 (6.42)
Obsessive-compulsive disorder	SVM	High beta PSD+FC	71.5 (12.9)	77.6 (25.3)	76 (23.1)	Alpha FC	75.2 (11.2)	66.7 (19.6)	91.5 (11.1)
	RF	Delta PSD	72.49 (8.05)	71.67 (19.6)	79 (16.47)	Alpha PSD	76.71 (18.89)	71.44 (24.94)	89 (15.06)
	EN	Delta PSD	70.73 (12.47)	69.33 (25.61)	80.5 (28.72)	Gamma FC	74.52 (18.43)	65.33 (22.1)	90 (21.6)
Addictive disorder	SVM	Theta PSD	74.3 (12.6)	80.1 (14.4)	67.4 (21.9)	Theta PSD	82.1 (11.4)	77 (15.6)	85.2 (16.7)
	RF	Delta PSD	78.01 (7.11)	70.96 (16.31)	85.33 (14.05)	Delta PSD	84.64 (7.63)	79.36 (10.89)	83.89 (12.97)
	EN	Beta PSD	80.62 (7.09)	74.12 (11.36)	86.33 (8.91)	Theta PSD	85.66 (5.22)	71.61 (12.45)	94.89 (5.4)
Trauma and stress-related disorder	SVM	Delta PSD	86.9 (9.4)	88.4 (10.4)	83 (18.7)	Whole PSD	90.7 (5.2)	90.6 (8.8)	84.1 (13.4)
	RF	Delta PSD+FC	89.86 (6.68)	86 (14.04)	89.94 (10.27)	Delta FC	92.28 (6.22)	94.78 (7.26)	84.26 (10.08)
	EN	Beta PSD+FC	86.51 (7.51)	80.89 (17.16)	86.03 (11.85)	Beta FC	91.21 (5.3)	86.44 (9.58)	90.64 (6.04)

Note. AUC = Area Under Curve, HC = Healthy Control, SVM = Support Vector Machine, RF = Random Forest, EN = Elastic Net, PSD = Power Spectrum Density, FC = Functional Connectivity, and IQ = Intelligence Quotient.

Table S2. Comparisons of AUC performance of classification models for specific psychiatric disorders distinguished from HCs

Main Caterory of disorder	ML method	Best feature	Predictor: EEG+age+sex+education AUC	Specificity	Sensitivity	Best feature	Predictor: EEG+age+sex+education+IQ r AUC	Specificity	Sensitivity
			Mean(SD)						
Depressive disorder	SVM	Delta PSD+FC	82.49 (7.21)	74.39 (14.76)	86.22 (12.26)	Delta FC	86.7 (8.7)	77.9 (10.6)	88.7 (11.2)
	RF	Delta PSD+FC	84.48 (6.87)	78.89 (11.97)	84.22 (16.36)	Gamma AB	86.9 (4.23)	79.45 (8.89)	90.56 (11.53)
	EN	Delta FC	86.18 (4.72)	72.84 (7.61)	93.67 (7.22)	Delta FC	87.91 (5.67)	80.82 (15.1)	91.44 (12.35)
Bipolar disorder	SVM	Whole PSD+FC	85.3 (11.75)	90.95 (12.98)	76.67 (16.13)	Alpha PSD+FC	87.6 (7.5)	84 (14.2)	87.6 (7.5)
	RF	Delta PSD+FC	81.58 (12.54)	86.43 (11.45)	77.78 (19.25)	Whole PSD+FC	89.5 (4.44)	90.95 (7.84)	84.33 (9.89)
	EN	Delta FC	88.08 (9.8)	92.86 (13.88)	79.33 (15.67)	Delta PSD+FC	92.13 (3.01)	90.71 (11.25)	85 (10.76)
Panic disorder	SVM	Whole FC	87.07 (11.12)	81.89 (14.62)	91 (15.56)	Theta PSD	87.3 (9.3)	94.6 (7.8)	79.3 (16.1)
	RF	Delta PSD+FC	85.95 (11.51)	79.33 (21.23)	90 (17.92)	Delta PSD+FC	89.57 (7.93)	89.22 (13.87)	85 (12.3)
	EN	Theta FC	89.91 (9.31)	92.56 (10.44)	88.33 (17.66)	Whole PSD	90.07 (5.32)	89.44 (11.04)	88 (11.46)
Social anxiety disorder	SVM	Theta PSD+FC	83.33 (15.26)	84 (15.54)	87.5 (10.87)	Theta PSD+FC	89.4 (10.5)	89.7 (9.4)	88 (16.9)
	RF	Gamma FC	90.51 (9.1)	85.33 (12.37)	91.5 (14.54)	Delta PSD	91.99 (7.71)	90 (16.33)	91 (11.74)
	EN	Delta PSD+FC	87.06 (13.06)	86.11 (15.78)	88 (19.32)	Theta FC	90.63 (8.51)	91.56 (6.5)	88 (13.98)
Alcohol use disorder	SVM	Delta FC	86.41 (9.77)	85 (15.74)	85.33 (8.71)	Alpha PSD	89.9 (7.2)	82.6 (16.7)	91.8 (6.4)
	RF	Delta FC	85.55 (6.88)	74 (22.32)	87.78 (13.33)	Alpha PSD	89.27 (7.59)	75.22 (15.94)	96.78 (7.38)
	EN	Whole PSD	87.11 (7.39)	82.67 (15.94)	87.67 (12.87)	Whole PSD	93.21 (6.31)	92.33 (7.45)	88.44 (13.62)
Behavioral addiction disorder	SVM	Delta PSD	74.17 (11.28)	67.89 (19.91)	74.17 (11.28)	Theta PSD	81.4 (9.7)	17.1 (80.44)	16.4 (83.67)
	RF	Beta PSD	79.31 (11.13)	73 (23.41)	83.67 (18.69)	Delta PSD	83.37 (7.78)	80.44 (13.8)	83.67 (20.14)
	EN	Theta PSD	79.35 (9.96)	80 (23.63)	77.22 (17.57)	Delta PSD	84.78 (8.85)	81.33 (13.87)	83.67 (14.34)
Posttraumatic stress disorder	SVM	Delta PSD	91.78 (12.18)	93.67 (8.92)	90 (14.14)	Delta PSD	93.6 (5.3)	88.3 (10.9)	96 (8.4)
	RF	Alpha PSD+FC	95.08 (6.47)	87.55 (15.6)	98.33 (5.27)	Beta PSD+FC	97.1 (8.1)	96.77 (5.19)	85 (15.8)
	EN	Beta PSD	94.38	92.66	92.66	Beta PSD	95.38	95.88	92

Main Caterory of disorder	ML method	Best feature	Predictor: EEG+age+sex+education AUC	Specificity	Sensitivity	Best feature	Predictor: EEG+age+sex+education+IQ r AUC	Specificity	Sensitivity
			Mean(SD)						
			(5.97)	(8.38)	(12.35)		(4.9)	(7.1)	(10.32)
Acute stress disorder	SVM	Whole PSD	84.39<br>(10.77)	90<br>(17.48)	81<br>(17.11)	Delta PSD	86<br>(10.9)	90<br>(12.9)	84.3<br>(17.9)
	RF	Whole PSD	86<br>(10.99)	92.5<br>(16.87)	79.88<br>(16.66)	Delta PSD+FC	89.03<br>(7.85)	97.5<br>(7.9)	80.11<br>(16.84)
	EN	Delta FC	89.71<br>(6.19)	1<br>(0)	78.11<br>(10.17)	Beta PSD+FC	92<br>(6.63)	95<br>(10.54)	89.44<br>(11.27)
Adjustment disorder	SVM	Theta PSD+FC	84.58<br>(7.93)	91.67<br>(13.61)	79.11<br>(13.13)	Gamma PSD	92.2<br>(7.1)	90<br>(12.9)	93.7<br>(7.1)
	RF	Theta PSD	87<br>(5.26)	90<br>(12.9)	86.33<br>(12.76)	High beta FC	94.65<br>(8.77)	96.66<br>(10.54)	92<br>(13.98)
	EN	Theta FC	88.08<br>(9.38)	84.16<br>(13.86)	90.33<br>(14.09)	Alpha FC	93.75<br>(7.31)	95<br>(10.54)	91.66<br>(13.42)

Note. AUC = Area Under Curve, HC = Healthy Control, SVM = Support Vector Machine, RF = Random Forest, EN = Elastic Net, PSD = Power Spectrum Density, FC = Functional Connectivity, and IQ = Intelligence Quotient.

Table S3. Mean region-wise coefficient of survived predictors for main category of psychiatric disorders

disorder	parameter	band	channel	survival number	mean	SD
**Schizophrenia disorder**						
	PSD	Alpha	C4	7	0.05	0.02
	PSD	Alpha	T5	8	0.01	0
	PSD	Alpha	Pz	7	-0.04	0.03
	PSD	Alpha	O2	10	0.02	0.01
**Mood disorder**						
	FC	Theta	FP1F8	10	0.02	0.01
	FC	Theta	FP1C3	10	-0.03	0.02
	FC	Theta	FP2F4	10	-0.04	0.01
	FC	Theta	FP2F8	10	0.01	0.01
	FC	Theta	F7T6	8	0.01	0.01
	FC	Theta	F3T3	8	-0.01	0.01
	FC	Theta	F3P4	9	0.03	0.01
	FC	Theta	FzF4	8	-0.01	0.01
	FC	Theta	FzT3	9	-0.02	0.01
	FC	Theta	FzC4	10	-0.01	0.01
	FC	Theta	C3O2	7	0.01	0
	FC	Theta	CzT5	9	-0.02	0.01
	FC	Theta	CzP4	7	0	0
	FC	Theta	T4P3	10	0.03	0.01
	FC	Theta	T5P3	10	-0.01	0
	FC	Theta	P4T6	10	-0.02	0
	FC	Theta	P4O2	9	-0.01	0.01
	FC	Theta	T6O1	10	0.02	0.01
**Anxiety disorder**						
	PSD	Delta	T3	8	-0.02	0.02
	PSD	Delta	T5	10	-0.08	0.02
	PSD	Delta	O2	10	-0.08	0.02
	PSD	Theta	Cz	10	0.03	0.01
	PSD	Theta	Pz	10	0.1	0.02

disorder	parameter	band	channel	survival number	mean	SD
	PSD	Alpha	T4	9	0.03	0.01
	PSD	Alpha	O2	10	-0.01	0
	PSD	Beta	FP2	8	-0.02	0.01
	PSD	Beta	C4	9	-0.03	0.02
	PSD	Beta	T4	9	-0.06	0.03
	PSD	Highbeta	Cz	10	-0.12	0.06
	PSD	Gamma	F8	10	0.12	0.02
	PSD	Gamma	T4	10	0.15	0.05
	PSD	Gamma	P4	7	0.13	0.07
**Obsessive-compulsive disorder**						
	FC	Gamma	FP1FP2	10	-0.01	0.01
	FC	Gamma	FP1F3	7	-0.01	0
	FC	Gamma	F3Fz	10	-0.02	0.01
	FC	Gamma	C4P4	10	0.05	0.01
**Addictive disorder**						
	PSD	Theta	FP1	10	-0.08	0.04
	PSD	Theta	F8	8	0.1	0.03
	PSD	Theta	C4	9	0.03	0.02
	PSD	Theta	T5	10	-0.06	0.02
**Trauma and stress-related disorder**						
	FC	Beta	FP1T3	9	-0.01	0.01
	FC	Beta	FP1T6	10	-0.04	0.02
	FC	Beta	FP2F4	8	-0.01	0
	FC	Beta	FzF4	7	-0.01	0.01
	FC	Beta	FzCz	10	-0.03	0.01
	FC	Beta	FzC4	9	-0.03	0.01
	FC	Beta	F8T6	7	-0.02	0.01
	FC	Beta	C3C4	10	0.02	0.01
	FC	Beta	C3T4	10	0.03	0.01
	FC	Beta	C3P3	10	0.02	0.01
	FC	Beta	C3Pz	10	0.03	0.01

disorder	parameter	band	channel	survival number	mean	SD
	FC	Beta	C3P4	7	0.01	0
	FC	Beta	T4O1	10	0.01	0.01
	FC	Beta	T5O1	10	-0.02	0
	FC	Beta	P4O2	7	-0.01	0.01
	FC	Beta	T6O2	10	-0.01	0.01

note. survived predictors = emerged significant over 7 time during 10-time cross-validation. PSD = Power Spectrum Density and FC = Functional Connectivity.

Table S4. Mean region-wise coefficient of survived predictors for specific psychiatric disorders

disorder	parameter	band	channel	survival number	mean	SD
**Depressive disorder**						
	FC	Delta	FP1T3	10	0.02	0.01
	FC	Delta	FP2F4	10	0.01	0
	FC	Delta	F3Fz	9	0	0
	FC	Delta	F3P3	8	-0.01	0.01
	FC	Delta	F3P4	10	-0.04	0.01
	FC	Delta	FzF4	10	0.01	0
	FC	Delta	FzCz	10	0.02	0
	FC	Delta	FzC4	9	0.01	0
	FC	Delta	F4F8	7	0	0
	FC	Delta	F4T4	10	0.01	0.01
	FC	Delta	C3Cz	10	0.01	0.01
	FC	Delta	C3T6	9	-0.01	0
	FC	Delta	C3O2	8	-0.01	0.01
	FC	Delta	T4O1	10	-0.03	0.01
	FC	Delta	T5P3	10	0.01	0
	FC	Delta	P4O2	10	0.01	0
	FC	Delta	T6O2	10	0.01	0
**Bipolar disorder**						
	PSD	Delta	F8	8	0.02	0.02
	PSD	Delta	O2	10	-0.04	0.02
	FC	Delta	FP1F8	7	-0.01	0.01
	FC	Delta	FP1T3	10	0.03	0.01
	FC	Delta	FP1Cz	7	0.02	0.01
	FC	Delta	FP2F4	10	0.01	0.01
	FC	Delta	F7Fz	10	0.01	0.01
	FC	Delta	F3Fz	10	0.03	0.02
	FC	Delta	F3P4	10	-0.05	0.02
	FC	Delta	F3O2	10	-0.03	0.01
	FC	Delta	FzC4	7	0.01	0.01

disorder	parameter	band	channel	survival number	mean	SD
	FC	Delta	F4C4	7	0.01	0.01
	FC	Delta	C3Cz	10	0.03	0.02
	FC	Delta	T5P3	10	0.01	0.01
	FC	Delta	P4T6	9	0.01	0.01
**Panic disorder**						
	PSD	Delta	T5	9	0.1	0.07
	PSD	Delta	P4	9	-0.05	0.02
	PSD	Delta	O2	10	0.09	0.03
	PSD	Theta	Pz	10	-0.12	0.03
	PSD	Alpha	T4	9	-0.03	0.01
	PSD	Alpha	O2	7	0.01	0.01
	PSD	Beta	FP2	7	0.04	0.02
	PSD	Beta	T3	7	0.03	0.02
	PSD	Beta	T6	9	0.02	0.01
	PSD	Highbeta	Cz	9	0.16	0.06
	PSD	Highbeta	C4	7	0.2	0.13
	PSD	Highbeta	O1	9	0.29	0.13
	PSD	Gamma	F8	10	-0.1	0.14
	PSD	Gamma	T4	10	-0.21	0.11
	PSD	Gamma	P4	10	-0.46	0.23
**Social anxiety disorder**						
	FC	Theta	FP2F4	10	-0.03	0.01
	FC	Theta	F7T3	9	-0.01	0.01
	FC	Theta	F3P3	10	0.03	0.01
	FC	Theta	F3O2	7	0.02	0.01
	FC	Theta	F4C4	7	-0.01	0.01
	FC	Theta	F8T4	9	-0.01	0
	FC	Theta	T4P3	10	0.01	0.01
	FC	Theta	T5P3	9	-0.01	0.01
**Alcohol use disorder**						
	PSD	Delta	F7	8	0.02	0.01

disorder	parameter	band	channel	survival number	mean	SD
	PSD	Delta	F8	8	0.01	0.01
	PSD	Delta	Cz	7	0.01	0.01
	PSD	Delta	T5	9	-0.03	0.02
	PSD	Alpha	FP1	8	-0.01	0.01
	PSD	Beta	FP1	10	-0.1	0.05
	PSD	Beta	T3	9	-0.05	0.02
	PSD	Beta	T5	7	-0.01	0.01
	PSD	Highbeta	T3	9	-0.16	0.09
	PSD	Highbeta	T4	10	0.21	0.13
	PSD	Gamma	F8	9	0.22	0.1
	PSD	Gamma	C4	9	0.21	0.08
	PSD	Gamma	Pz	9	0.19	0.21
**Behavioral addiction disorder**						
	PSD	Delta	Fz	10	-0.05	0.01
	PSD	Delta	F8	10	0.03	0.01
	PSD	Delta	T5	10	-0.06	0.03
	PSD	Delta	P4	9	0.06	0.03
	PSD	Delta	T6	7	0.03	0.02
	PSD	Delta	O2	10	-0.04	0.02
**Posttraumatic stress disorder**						
	PSD	Beta	FP1	8	0.04	0.01
	PSD	Beta	T5	10	0.12	0.06
**Acute stress disorder**						
	PSD	Beta	FP1	10	0.08	0.02
	PSD	Beta	T5	9	0.04	0.02
	FC	Beta	FP2F3	10	-0.02	0.01
	FC	Beta	FzF4	10	-0.03	0.02
	FC	Beta	FzCz	8	-0.02	0.02
	FC	Beta	C3Cz	10	0.03	0.02
	FC	Beta	C3C4	10	0.05	0.01
	FC	Beta	C3P3	10	0.03	0.01

disorder	parameter	band	channel	survival number	mean	SD
	FC	Beta	T5O1	7	-0.01	0
	FC	Beta	T6O2	9	-0.01	0.01
**Adjustment disorder**						
	FC	Alpha	FP2Fz	7	-0.02	0.01
	FC	Alpha	FzCz	10	-0.02	0.02
	FC	Alpha	F8T4	8	-0.01	0.01
	FC	Alpha	C3T5	7	-0.01	0.01
	FC	Alpha	C4P3	8	0.02	0.01
	FC	Alpha	T4P4	10	0.03	0.01
	FC	Alpha	P4T6	8	-0.02	0.01
	FC	Alpha	P4O2	8	-0.01	0.01

note. survived predictors = emerged significant over 7 time during 10-time cross-validation. PSD = Power Spectrum Density and FC = Functional Connectivity.

Table S5. Predicting outcomes for between psychiatric disorder classification models

	Feature (entire)	AUC Mean (SD)	Sens	Spec	Feature (best)	AUC Mean (SD)	Sens	Spec	*P* value (best)
SchizophreniaVS Mooddisorder	entire	62.55 (7.39)	58.10 (21.19)	73.54 (16.25)	alpha AB	68.08 (7.22)	58.33 (12.16)	80.88 (8.56)	0.002
Schizophrenia VS Depressive disorder	entire	58.96 (8.56)	79.01 (20.47)	52.26 (28.19)	theta COH	68.69 (12.67)	72.04 (20.67)	68.36 (21.04)	0.002
Schizophrenia VS Bipolar disorder	entire	57.18 (17.61)	63.78 (30.03)	70.23 (30.59)	delta AB+COH	67.83 (13.66)	59.92 (18.91)	85.00 (15.63)	0.016
Depressive disorder VS Bipolar disorder	entire	50.77 (11.63)	64.47 (32.5)	58.57 (33.37)	delta AB+COH	63.55 (6.95)	63.86 (23.24)	75.95 (21.77)	0.039
Panic disorder VS Social anxiety disorder	entire	54.03 (18.55)	85.50 (19.21)	52.33 (28.41)	whole AB	70.46 (20.91)	85.00 (18.40)	71.33 (30.35)	0.027

Note: age, sex, education, and IQ were included in the model. Permutation test was conducted in order to assess the significance of each of the best EN models. Abbreviations: EN = elastic net, AB = power spectrum density, COH = coherence, FC = functional connectivity, IQ = intelligence quotient.
